# Size and Shape Directed Novel Green Synthesis of Plasmonic Nanoparticles Using Bacterial Metabolites and Their Anticancer Effects

**DOI:** 10.3389/fmicb.2022.866849

**Published:** 2022-04-11

**Authors:** Snehal Patil, Murali Sastry, Atul Bharde

**Affiliations:** ^1^Department of Microbiology, Savitribai Phule Pune University, Pune, India; ^2^Department of Materials Science and Engineering, Monash University, Clayton, VIC, Australia

**Keywords:** nanoparticle biosynthesis, plasmonic nanoparticles, anisotropic nanoparticles, nanomedicine, cancer nanotechnology

## Abstract

The growing need for developing new synthesis methods of plasmonic nanoparticles (PNPs) stems from their various applications in nanotechnology. As a result, a variety of protocols have been developed for the synthesis of PNPs of different shapes, sizes, and compositions. Though widely practiced, the chemical synthesis of PNPs demands stringent control over the experimental conditions, often employs environmentally hazardous chemicals for surface stabilization, and is frequently energy-intensive. Additionally, chemically obtained PNPs require subsequent surface engineering steps for various optoelectronic and biomedicine applications to minimize the toxic effects and render them useful for targeted drug delivery, sensing, and imaging. Considering the pressing need to develop environmentally-friendly technology solutions, “greener” methods of nanoparticle synthesis are gaining importance. Here, we report on the biological synthesis of plasmonic nanoparticles using bacterial metabolites. A peptide-based siderophore pyoverdine and a blue-green pigment pyocyanin obtained from a marine strain of *Pseudomonas aeruginosa* rapidly produced plasmonic nanoparticles of gold and silver in an aqueous environment. The morphology of plasmonic nanoparticles could be modulated by tuning the concentration of these metabolites and the reaction time. The exposure of pyoverdine to chloroauric acid resulted in anisotropic gold nanoparticles. On the other hand, pyocyanin produced a highly monodispersed population of gold nanoparticles and anisotropic silver nanoparticles. Biologically obtained gold and silver nanoparticles retained pyoverdine and pyocyanin on the nanoparticle surface and were stable for an extended period of time. The biologically obtained gold and silver plasmonic nanoparticles displayed potent anticancer activities against metastatic lung cancer cells. Biogenic nanoparticles were rapidly internalized by cancer cells in high quantity to affect the cellular organization, and karyoplasmic ratio, indicating the potential of these nanoparticles for cancer nanomedicine.

## Introduction

Developing new protocols for the synthesis of metallic nanoparticles of different sizes and shapes constitutes an ever-growing field of nanoscience. Among various metallic nanoparticles, plasmonic nanoparticles (PNPs) of gold and silver show exciting optoelectronic properties such as wavelength selective plasmon absorption resonances, conductivity, and photothermal/photodynamic effect (Jiang et al., 2018). These properties of PNPs depend on their size and shapes and have been exploited for numerous applications in catalysis, electronics, non-linear optics, single-electron transistors, sensors, and biomedicine (Sotiriou, 2013; Barbillon, 2019; Garcia-Lojo et al., 2019). Therefore, it is highly desirable to produce gold and silver PNPs with different morphology and size, tuned to their applications. The synthesis of gold and silver PNPs with myriad shapes and sizes is extensively developed using several chemical or physical synthesis protocols (Lu et al., 2009; Abid et al., 2021; Hopper et al., 2022). Although widely practiced as a primary choice of PNPs synthesis, chemical methods could exert potential health hazards like carcinogenicity, toxicity, and environmental pollution. The prominent toxicity concerns are due to the hazardous reducing agents, organic solvents, and stabilizing surfactants used in the synthesis and stabilization of PNPs (Bahadar et al., 2016; Sukhanova et al., 2018). These toxic reagents limit the clinical and biomedical applications of PNPs. Equally, physical methods, although do not employ toxic chemicals, are energy-intensive and often require sophisticated tools for the preparation of PNPs (Ramanath et al., 2020).

Conversely, the biological synthesis of PNPs occurs in aqueous environments at ambient conditions of temperature and pressure. Therefore, besides being environmentally friendly and economical, biological synthesis may have advantages for PNP applications when an aqueous environment is desired. Biological synthesis can clearly offer reliable, clean, biologically appropriate, and environmental-friendly techniques to synthesize nanoparticles. This is because it can proceed without the use of non-toxic agents, additional stabilizers and reducing agents, and require low energy expenditure. Ecological safety is the key advantage of the biological synthesis method over chemical and physical protocols. Indeed, research from the past two decades has demonstrated the promise of biological synthesis of inorganic materials and its gaining importance due to the environmental hazards caused by counterpart chemical synthesis methods (Li et al., 2011; Bansal et al., 2012). Many biological systems exert exquisite control over the formation of the metal nanoparticles through concerted mechanisms.

The biological synthesis of nanoparticles, including PNPs of gold and silver, is reported using numerous methods. They include the use of prokaryotic micro-organisms like bacteria and simple eukaryotic fungi and algal cells (Thakkar et al., 2010; Iravani, 2014; Grasso et al., 2019; Zhang et al., 2020). Several bacterial species are explored for the size and shape-controlled synthesis of gold and silver PNPs (Sastry et al., 2005; Zhang et al., 2020). Additional to excellent optical properties, bacterially synthesized PNPs show high antimicrobial activities (Iravani, 2014; Gahlawat and Roy Choudhury, 2019). Besides micro-organisms, a variety of plant extracts, rich in alkaloids and phenolic compounds, are extensively employed for PNP synthesis. Most of the plant-extract based methods provide a high yield of PNPs, while some are useful for the size and shape-controlled synthesis (Chandran et al., 2006; Chung et al., 2016; Singh et al., 2016). Several studies have also demonstrated the use of proteins and enzymes for the green synthesis of PNPs *in vitro* (Ulijn and Jerala, 2018). Mostly, the synthesis of oxide nanoparticles such as silica and magnetite, and sulfide nanoparticles such as CdS and ZnS is successfully achieved by this approach. The synthesis of silica and silicones is extensively investigated using silicateins, silaffins, and silica precipitating peptides (Lechner and Becker, 2015; Ulijn and Jerala, 2018). Similarly, *in vitro* synthesis of magnetic iron oxide nanoparticles (NPs) is reported using magnetosome proteins isolated from magnetotactic bacteria (Nudelman et al., 2016). Parallel approaches are used for the synthesis of gold PNPs using dodecapeptides derived from the phage display library (Egorova et al., 2020). A small peptide isolated from a combinatorial phage display peptide library associated with silver binding and reduction is used to synthesize silver nanoparticles with a variety of crystal morphologies (Slocik et al., 2005).

Although living microorganisms are extensively explored for the biosynthesis of nanomaterials, their metabolic products besides proteins, are not much explored in this regard. Here, as a pursuit of newer green chemistry methods of NP synthesis, we investigated the potential of bacterial primary and secondary metabolites for the synthesis of anisotropic gold and silver PNPs and further explored their antiproliferative properties against metastatic cancer cells. The bacterial genus Pseudomonas consists of many species known to produce small peptides as growth-associated metabolites. Pyoverdine and cyclodipeptides are prominent examples from *Pseudomonas aeruginosa* and help bacteria to chelate iron and facilitate quorum sensing (Visca et al., 2007). Additionally, *P. aeruginosa* synthesizes water diffusible, blue-green colored pigment, pyocyanin, which shows antibacterial properties (Crosa and Walsh, 2002; Özyürek et al., 2016). In this work we demonstrated rapid, synthesis of anisotropic gold and silver PNPs using pyoverdine and pyocyanin obtained from an indigenously isolated strain of *P. aeruginosa* (Mavrodi et al., 2001). Pyoverdine and pyocyanin show excellent redox properties, and therefore can reduce gold and silver ions to yield respective NPs. Additionally, these small molecules possess high-affinity functional groups that can bind to NPs surfaces to cap and stabilize them.

## Materials and Methods

### Materials

Chloroauric acid, silver nitrate, Ethyl acetate, Penicillin and streptomycin, 3-(4,5-dimethylthiazol-2-yl)-2,5- diphenyltetrazolium bromide (MTT) cell survival assay reagents were purchased from Sigma-Aldrich (St. Louis, MO, United States). Luria broth, King’s B Medium was from Hi-media Laboratories (Mumbai, India). Dulbaco’s modified eagle medium (DMEM), fetal bovine serum (FBS), anti EEA1 rabbit monoclonal antibody, goat anti-rabbit Alexa fluor 488 secondary antibody, and phalloidin-Alexa 488 were purchased from Life Technologies (Carlsbad, CA, United States).

### Isolation and Purification of Pyoverdine and Pyocyanin From *Pseudomonas aeruginosa* 25W

The siderophore pyoverdine was isolated from *P. aeruginosa* 25W using a previously reported method (Özyürek et al., 2016). Briefly, the bacterial seed culture was grown in an Luria-Bertani (LB) medium for a period of 12 h. The actively growing seed culture (O.D. adjusted to 0.1) was then inoculated in 100 ml of King’s B medium in a 500 ml Erlenmeyer flask for the production of pyoverdine. The flask was incubated on the shaker (150 rpm) for 24 h at 30°C. Next, pyoverdine was isolated from the aqueous medium by the solvent extraction method described below. The supernatant containing pyoverdine was collected by centrifugation (at 5,500 × *g*) and the pellet containing the bacterial cells was discarded. The supernatant was acidified with concentrated hydrochloric acid until the pH of the solution became 2. The acidified supernatant was mixed with ethyl acetate in a 1:1 ratio and incubated overnight. The organic layer was separated from the aqueous layer, evaporated, and dried, and the powdered residue of pyoverdine was dissolved in 10 ml of 100% methanol.

Pyocyanin was isolated from *P. aeruginosa* using a broth culture method as described previously (Crosa and Walsh, 2002). Freshly inoculated bacterial culture was grown and propagated in 100 ml LB medium in a 500 ml of Erlenmeyer flask. The flask was incubated at 30°C on the rotary shaker (200 rpm) and incubated for 72 h until the bacterial growth was in the late stationary phase when the medium showed dark green color. The bacterial biomass was then separated by centrifugation at 2,500 × *g* for 10 min. The colored supernatant was isolated and acidified with concentrated HCl, mixed with chloroform, and subsequently treated with the acidified and neutral water that allowed separation of the blue-green form of pyocyanin in chloroform from other impurities. After five such conversions, pyocyanin was crystallized by evaporation of chloroform and dried to the powder form.

The purity of pyoverdine and pyocyanin was assessed by thin-layer chromatography (TLC) using a Silicagel 60F254 TLC plate (Merck, Germany). The plates were developed using a chloroform-acetic acid-ethanol (90:5:2.5 vol/vol/vol) solvent system and then developed using a 0.1 mm acidified FeCl_3_ solution for the detection of pyoverdine. Pyocyanin was detected using a chloroform-acetic acid-methanol (90:5:2.5 vol/vol/vol) solvent system and visualizing the plates under ultraviolet (Uv) light exposure (λ 365 nm). The Rf values for pyoverdine and pyocyanin were calculated by measuring the distance traveled by pyoverdine and pyocyanin to their respective solvent fronts (Hoegy et al., 2014).

### Biological Synthesis of Pv-PNPs and Py-PNPs

For the biological synthesis of Pv-PNPs and Py-PNPs, either 0.01 or 0.1 mg/ml of pyoverdine and pyocyanin were mixed and reacted separately with the 10 ml aqueous solutions of chloroauric acid (HAuCl_4_) and AgNO_3_ at a final concentration of 10^–3^ M respectively. The reaction was continued for 1 h, and the formation of Pv-PNPs and Py-PNPs was confirmed visually as a color change of the reaction solution. Pv-PNPs and Py-PNPs were subsequently characterized by UV-Vis spectroscopy, Transmission electron microscopy (TEM), and Fourier transformed infrared spectroscopy (FTIR).

### Characterization of Pv-PNPs and Py-PNPs

The formation of biogenic Pv-PNPs (Pv-AuNPs and Pv-AgNPs) and Py-PNPs (Py-AuNPs and Py-AgNPs) was monitored by recording the Uv-Vis absorption spectra on a Uv-Vis-NIR spectrophotometer (JASCO, Tokyo, Japan). The kinetics of Au and AgNPs synthesis was studied by recording the spectra at every 15 min intervals for 1 h, after initiation of the reaction, at a scan rate of 1 nm/s. The morphology of Pv-PNPs and Py-PNPs was studied with TEM. TEM imaging was performed with a Jeol 1400 model (Jeol corporation, Japan) operating at 120 kv accelerating voltage and equipped with a charge coupled device (CCD) camera (Gatan Incorporation, United States). Samples for TEM analysis were drop-cast from diluted Pv-PNPs and Py-PNPs solutions onto carbon-coated TEM grids and allowed to air dry. FTIR analysis was performed to confirm the presence of pyoverdine and pyocyanin on Au and AgNPs surface and identify specific functional groups involved in the NP formation and stabilization process. For the FTIR analysis, respective Pv-PNPs and PyPNPs were purified by repetitive centrifugation, resuspended in deionized water, and powdered by lyophilization. Pv-PNPs, pyoverdine, Py-PNPs, and pyocyanin were mixed individually with the infrared (IR) grade potassium bromide (KBR) (Sigma, United States) to obtain their pellets. The spectra were recorded in attenuated total reflection (ATR) mode with an FTIR spectrophotometer (Perlin-Elmer, United States) in the range of 400 cm^–1^–4,000 cm^–1^ at a scan rate of 4 cm^–1^/s. The vibration patterns were analyzed according to a previous report.

### Effect of Pv-PNPs and Py-PNPs on the Survival of Cancer Cells

To determine the effect of Pv-PNPs and Py-PNPs on the survival of cancer cells, the viability of A549 lung cancer cells (non-small cell lung cancer cells) was determined using MTT assay (Kumar et al., 2018). The A549 cell line was obtained from an American-type culture collection, revived, and maintained according to the supplier’s instructions. For cell viability assay, cells were seeded in tissue culture grade 96 well-plates (Eppendorf, Germany) at a seeding density of 10^4^ cells/cm^2^ and grown for 24 h in DMEM supplemented with 5% FBS and 1% antibiotics (penicillin 100 units, streptomycin 10 ug/ml) at 37°C and 5% carbon dioxide (CO_2)_ saturation. Next, cells were individually treated with different concentrations (0.1 μg/ml–1 mg/ml) of PvPNPs (Pv-AuNPs and Pv-AgNPs) and Py-PNPs (Py-AuNPs and Py-AgNPs) for 24 h. Subsequently, 10 μl of MTT reagent (Sigma, United States) was added to each well (final concentration 0.45 mg/ml). After 1 h of incubation, an equal volume of 10% dimethylsulfoxide (DMSO) was added to each well to allow the solubilization of formazan crystals. Absorbance at 595 nm was recorded using a microtiter plate reader (Molecular Device, USA). For cell viability, the optical density (OD) of each sample was compared with the control (Cells without Pv-PNPs or Py-PNPs) and% cell survival was calculated according to the formula

% Cell survival = Mean OD of sample/Mean OD of blank × 100

IC**_50_** values were obtained by plotting the percent cell survival against the NP concentration and fitting the resulting plot with a biphasic dose-response model (Sebaugh, 2011).

### Determination of Karyoplasmic Ratio of Cancer Cells

Karyoplasmic ratio (nuclear to cytoplasmic ratio) was determined for control, Pv-AuNPs, and Pv-AgNPs treated A549 cells. A549 cells were treated with Pv-PNPs for 12 h followed by washing with 1× phosphate buffered saline (PBS). Actin cytoskeleton distribution was determined by labeling the cells with phalloidin-Alexa fluor 488 dye. The control and Pv-PNPs treated cells were fixed and incubated with phalloidin Alexa 488 (1:300 dilution) overnight at 4°C. Afterward, cells were treated with 1 μm DAPI to stain the nucleus for 15 min, washed, and mounted on a microscope glass slide. Cells were imaged on a Nikon Ti2U fluorescence microscope (Nikon Corporation, Japan) equipped with a motorized stage and an scientific complementary metal-oxide-semiconductor (sCMOS) camera, using appropriate excitation and emission filters. Cytoplasmic and nuclear volumes were obtained by taking optical sections of cells and recording them as a volume stack (z stack) using the NIH ImageJ software. The karioplasmic ratio was computed from the nuclear and cytoplasmic volumes obtained from 50 individual cells for the control and Pv-PNPs treatment each. Statistical significance was recorded as a *P*-value, obtained by performing a non-parametric unpaired *t*-test with GraphPad Prism, San Diego, CA, United States software (version 8).

### Imaging Internalization of Pv-PNPs and Py-PNPs in Early Endosomes in Cancer Cells

To evaluate the endosomal localization of Pv-PNPs and Py-PNPs, A549 cells were seeded on 12 mm coverslips, grown in DMEM with 5% FBS overnight, before incubating with 10 μg/ml of Pv-AuNPs and Py-AuNPs respectively for 15 min at 37°C. Afterward, cells were washed with 1× PBS, fixed, and immunostained with anti EEA1 rabbit antibody (1:200 dilution), and detected with goat antirabbit secondary antibody conjugated to Alexa fluor 488 (1:700 dilution). Early endosomal localization of Py-AuNPs and Pv-AuNPs was imaged with Nikon A1R confocal laser scanning microscope (CLSM) (Nikon Corporation, Tokyo, Japan) equipped with a 60 × 1.43 NA oil immersion plan apochromat objective (Nikon Corporation, Tokyo, Japan) and using appropriate excitation and emission filters. To determine the presence of Pv-PNPs in early endosomal vesicles, a colocalization analysis between Pv-PNPs and EEA1 signal was performed. The colocalization index was determined using a JACoP plugin in NIH ImageJ software and expressed as Pierson’s cross-correlation coefficient.

## Results and Discussion

### Isolation and Purification of Pyoverdine and Pyocyanin From *Pseudomonas aeruginosa* 25W

The synthesis of siderophore by *P. aeruginosa* is induced by iron limiting conditions, which are generally developed in the stationary phase of bacterial growth due to the depletion of nutrients in the growth medium. The growth of *P. aeruginosa* in King’s B medium promotes the synthesis of pyoverdine. Pyoverdine was secreted extracellularly in the surrounding medium as indicated by the yellow–green color of the medium. Bacterial cell-free supernatant when acidified with concentrated HCl became pale brown in the color due to the presence of pyoverdine. The acidified supernatant was mixed with ethyl acetate for extended time. The organic layer showed a brown color due to the presence of pyoverdine, which was later isolated by evaporation of ethyl acetate. The presence of pyoverdine was indicated by the UV-Vis spectroscopic analysis, where pyoverdine showed a strong absorption at 405 nm (Supplementary Figure 1C). The purity of pyoverdine analyzed with TLC showed the presence of a single spot, after the development of the plate with acidified FeCl_3_ (Supplementary Figure 1A), as pyoverdine forms a highly stable brown colored complex with ferric ions under acidic conditions. The R_*f*_ values calculated for pyoverdine (0.43) was consistent with the reported value (Hoegy et al., 2014).

*Psuedomonas aeruginosa* synthesizes blue-green pigment pyocyanin, which could be visually detected by a greenish tint associated with the LB media after 48 h of bacterial growth. The presence of pyocyanin was indicated by UV-Vis spectroscopic analysis, where a strong absorption at 360 nm was observed. The TLC analysis indicated the presence of a single bright-blue spot after irradiation with the UV light (Supplementary Figure 1B). The R_*f*_ value calculated for pyocyanin was 0.62 which strongly correlated with the reported Rf value for pyocyanin (Hoegy et al., 2014). Pyocyanin showed a strong absorption profile with two absorption maxima centered at 330 and 370 nm (Supplementary Figure 1D) that is characteristic of a phenazine ring system.

### Synthesis and Characterization of Pv-PNPs

#### Synthesis and Characterization of Pv-AuNPs

**FIGURE 1:**
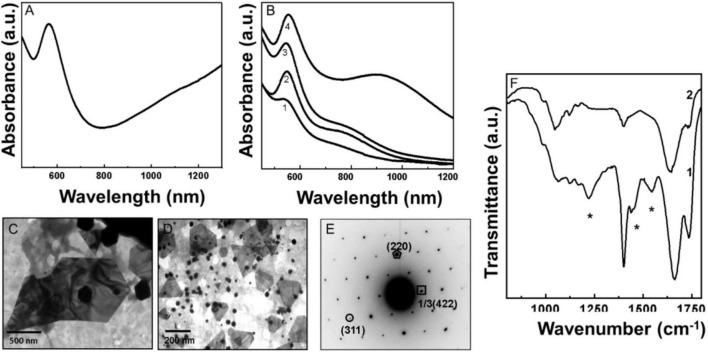
Uv-Vis spectroscopy, transmission electron microscopy (TEM), and Fourier transformed infrared spectroscopy (FTIR) analysis of biologically synthesized pyoverdine gold nanoparticles (Pv-AuNPs). **(A)** The Uv-Vis spectrum of Pv-AuNPs obtained after 60 min of reaction. **(B)** Kinetics of Pv-AuNPs formation followed by Uv-Vis absorption spectroscopy. Number 1–4 corresponds to 15, 30, 45, and 60 min of reaction. **(C,D)** TEM analysis of Pv-AuNPs showing flat hexagonal and triangular nanoparticles. **(E)** Selected area diffraction (SAED) pattern obtained from the triangular nanoparticles (NPs) shown in **(D)**. The numbers in parenthesis indicate the respective crystal planes corresponding to face centered cubic (FCC) gold. **(F)** FTIR spectra recorded from pyoverdine before (curve 1) and after (curve 2) the formation of Pv-AuNPs showing absence of various band vibrations belonging to functional groups (shown as *) in pyoverdine after NPs formation.

After the addition of pyoverdine to 10^–3^ M HAuCl_4_, the original yellow color of the reaction solution gradually changed to a dark red after1 h of reaction, indicating the formation of gold nanoparticles (Pv-AuNPs). Figure 1A shows Uv-Vis spectra of gold nanoparticles synthesized using 0.1 mg/ml solution of pyoverdine. A strong absorption band at 550 nm is due to the transverse surface plasmon oscillations and indicated the formation of gold nanoparticles. The transverse surface plasmon resonance (SPR) band was accompanied by absorption in the near-infra-red (NIR) region of the electromagnetic spectrum. The NIR absorption was due to the in-plane collective plasmonic oscillations indicating the anisotropic nature of gold particles (Hua et al., 2015). Figure 1B shows Uv-Vis spectra as a function of the time of reaction between the aqueous solution of gold ions and pyoverdin. The SPR band recorded after 15 min of reaction shows the presence of absorption at 550 nm indicating the predominance of the spherical AuNPs. This SPR band gradually showed an increase in the intensity with time and is accompanied by the in-plane SPR centered at 800 nm. The in-plane SPR increased in intensity with time and showed a gradual red shift after 60 min of reaction (curve 4, Figure 1B).

Representative TEM images of pyoverdine reduced gold ions solution after 60 min of reaction showed several nanoparticles with triangular and hexagonal morphology. The edge length of the triangular and hexagonal particles varied between 50 nm and 2 μm, depending on the concentration of pyoverdine used for the reduction of chloroaurate ions. At a higher concentration (0.1 mg/ml), flat triangular and hexagonal particles with an edge length between 50 nm and 1 um were predominantly observed (Figures 1C,D). However, at a lower concentration of pyoverdine (0.01 mg/ml), particles with the edge length between 500 nm to 2 μm dominated the NP population (Supplementary Figure 2). A number of small spherical AuNPs with a mean diameter of 20 nm were also observed along with flat triangular and hexagonal particles. Figure 1E shows the selected area diffraction pattern (SAED) obtained from one of the triangular AuNPs shown in Figure 1D. The SAED suggested that each triangular particle is a single crystal and the diffraction pattern was indexed on the basis of the face centered cubic (FCC) structure of gold. The corresponding *d* values for the respective crystal planes are as follows: 2.04 (200), 1.45 (220), 1.25 (311) (*JCPDS—International Center for Diffraction Data PCPDFWIN* version 1.30, 04-0784 for gold).

Fourier transformed infrared spectroscopy measurements were performed to identify the functional groups of pyoverdine involved in the reduction of gold ions and capping to AuNPs surface for their stabilization. Figure 1F shows the FTIR spectra of purified pyoverdine (curve 1) and pyoverdine—chloroaurate ions reaction mixture after the synthesis of gold nanoparticles (curve 2). A prominent absorption band at 1,144 cm^–1^ originated due to the stretching vibrations of C–N bonds, which was not observed in the spectrum corresponding to pyoverdine—chloroaurate ion reaction mixture after the formation of gold nanoparticles (curve 2, Figure 1F). The complete disappearance of a small vibrational band at 1,544 cm^–1^, assigned to amide II (N–H) stretching vibration from Pv-AuNPs, indicated that it could be involved in the formation of gold nanoparticles. This observation is consistent with the disappearance of the C–N bond absorption and supports the idea that amide or amine groups could be responsible for the reduction of chloroaurate ions or the capping on AuNPs surface. The presence of a weak absorption band centered at 1,438 cm^–1^ in curve 1 could be assigned to the CH_2_ group stretching vibrations that were not observed after the reduction of chloroaurate ions and Pv-AuNPs formation (curve 2, Figure 1F). The strong absorption centered at 1,663 cm^–1^ due to amide I (C = O) vibrational in pyoverdine (curve 1, Figure 1F) was shifted to lower wavenumber at 1,642 cm^–1^ (curve 2, Figure 1F) after the formation of Pv-AuNPs. This observation further suggests that amine or amide groups play an important role in the formation of AuNPs. A broad and strong absorption band at 3,140 cm^–1^ could be assigned either to the stretching vibrations of amine or hydroxyl groups present in pyoverdine as shown in spectrums 1 and 2 in Figure 1F. The absorption band at 3,140 cm^–1^ observed in curve 1 in Figure 1F, was considerably shifted to 3,400 cm^–1^ after the formation of gold nanoparticles (curve 2, Figure 1F). This shift could be because of the interaction of amine groups with the AuNPs surface. The disappearance or shift in amide stretching vibrations strongly suggested that this group of pyoverdine is possibly responsible for the synthesis of gold nanoparticles, their stabilization, or both. Supplementary Table 1 summarizes the vibration frequencies assigned for FTIR spectra shown in Figure 1F.

#### Synthesis and Characterization of Pv-AgNPs

**FIGURE 2:**
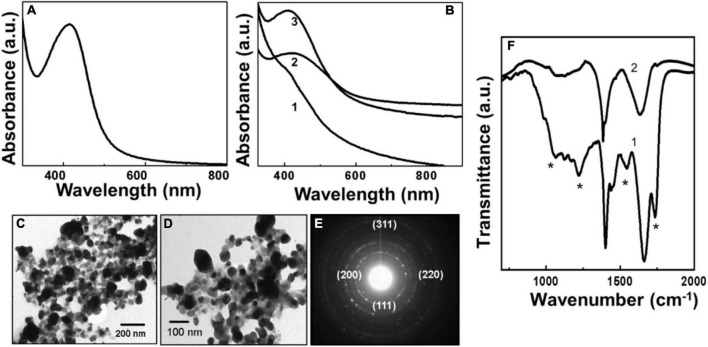
Uv-Vis spectroscopy, transmission electron microscopy (TEM), and Fourier transformed infrared spectroscopy (FTIR) analysis of biologically synthesized pyoverdine silver NPs (Pv-AgNPs). **(A)** The Uv-Vis spectrum of Pv-AgNPs synthesized after 60 min of reaction between pyoverdine and silver ions. **(B)** Pv-AgNPs formation kinetics investigated by Uv-Vis absorption spectroscopy. Number 1-3 corresponds to 15, 30, and 60 min of reaction. **(C,D)** TEM analysis of Pv-AgNPs showing nanoparticles of 20–30 nm diameter and irregularly shaped aggregates of 50–100 nm. **(E)** Selected area diffraction pattern (SAED) pattern obtained from Py-AgNPs shown in **(D)**. The numbers in parenthesis indicate the respective crystal planes corresponding to FCC silver. **(F)** FTIR spectra recorded from pyoverdine before (curve 1) and after (curve 2) the formation of Pv-AgNPs. The * sign indicates vibrations from pyoverdine which are significantly affected after the formation of pv-AgNPs.

Like the synthesis of AuNPs, pyoverdine could reduce silver ions and form AgNPs. Figure 2A shows the Uv-Vis spectrum of AgNPs formed after the complete reduction of silver ions with 0.1 mg/ml aqueous solution of pyoverdine. A strong SPR band centered at 425 nm indicated the presence of spherical AgNPs. Figure 2B shows the kinetics of Pv-AgNPs formation after reacting pyoverdine with 10^–3^M AgNO_3_ solution. Following the addition of 0.1 mg/ml pyoverdine to aqueous silver ions and subsequent incubation for 15 min, a weak absorption hump at 440 nm was observed (curve 2, Figure 2B). This absorption originated due to the SPR of silver nanoparticles and is the reason for the bright yellow color of the solution of silver nanoparticles. The absorption at 440 nm gradually increased in intensity with time and showed a slight blue shift to center at 425 nm, as indicated by curves 2 and 3 in Figure 2B, which corresponds to the Uv-Vis absorption spectra after 30 and 60 min of reaction. After 60 min of reaction, no appreciable rise in the absorption intensity at 425 nm was observed indicating the completion of silver ion reduction and Pv-AgNPs formation.

Figures 2C,D shows the TEM images of silver nanoparticles synthesized by reaction between aqueous silver ions and pyoverdine after 1 h reaction. A large number of Pv-AgNPs were observed to be distributed throughout the grid surface. The nanoparticles were highly irregular in shape and size with a broad particle size distribution. The AgNPs were predominantly in the size range of 50–100 nm aggregates, with occasional clusters greater than 100 nm. Pv-AgNPs were not regularly dispersed and showed aggregates consisting of smaller particles. The nanoparticles are observed to be embedded in a continuous matrix consisting of the organic matter as indicated by the lighter contrast at the periphery of silver nanoparticles (Figure 2D). Therefore, the overall structure appeared as an interconnected network with a high degree of irregularity in the arrangement of NPs. Unlike for AuNPs, pyoverdine concentration did not have an effect on the morphology of AgNPs. SAED analysis obtained from Pv-AgNps showed well-defined diffraction rings indicating the crystalline nature of silver nanoparticles (Figure 2E). The diffraction pattern was readily indexed on the basis of the FCC structure of silver. The corresponding *d* values for the respective crystal planes are as follows: 2.372 [111], 2.05 [200], 1.44 [220], 1.24 [311] (*JCPDS—International Center for Diffraction Data PCPDFWIN* version 1.30, 04-0783 for silver).

Figure 2F shows the FTIR spectra obtained from pyoverdine molecules before and after reaction with the silver ions showing significant variation in the vibration bands due to the reduction of silver ions and the formation of Pv-AgNPs. Similar to Pv-AuNPs, a prominent band at 1,563 cm^–1^ due to the amide I vibrational band in pyoverdine (curve 1, Figure 2F) was shifted to the lower wavenumber at 1,533 cm^–1^ (curve 2, Figure 2F) after reaction with silver ions and Pv-AgNPs formation. Two distinct bands in 1,530 to 1,670 cm^–1^ region were assigned to vibrations of amide I and II originating from the amide bonds of the peptide chain of pyoverdine. The amide II vibration band at 1,670 cm^–1^ arising from the pyoverdine molecule (curve2, Figure 2F) was not observed after Pv-AgNPs formation. The absence of the amide II band along with the shift of the amide I band indicated that the peptide chain may be involved in the reduction of silver ions or stabilization of Py-AgNPs. The shift in the amide I band vibration could be due to the binding of –CO– group of pyoverdine on the surface of PyAgNPs. Supplementary Table 1 summarizes the vibration frequencies assigned for FTIR spectra shown in Figure 2F.

### Synthesis and Characterization of Py-PNPs

#### Synthesis and Characterization of Py-AuNPs

**FIGURE 3:**
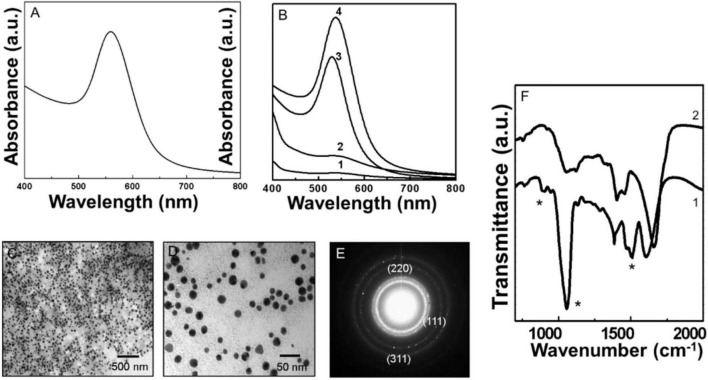
Uv-Vis spectroscopy, transmission electron microscopy (TEM), and Fourier transformed infrared spectroscopy (FTIR) analysis of biologically synthesized Py-AuNPs. **(A)** The Uv-Vis spectrum of Py-AuNPs synthesized after 60 min of reaction between pyocyanin and silver ions. **(B)** Pv-AuNPs formation kinetics followed by Uv-Vis absorption spectroscopy. Curves 1 to 4 represent time intervals of 15, 30, 45, and 60 min respectively. **(C,D)** Representative TEM images of Pv-AuNPs showing the presence of monodisperse spherical nanoparticles. **(E)** SAED pattern obtained from Pv-AuNs shown in the TEM images. **(F)** FTIR spectra recorded from pyocyanin before (curve 1) and after (curve 2) the formation of Py-AuNPs. The * sign indicates vibrations from pyoverdine which are significantly affected after the formation of py-AuNPs.

The reduction of gold ions and formation of AuNPs due to pyocyanin was evaluated using Uv-Vis spectroscopy as shown in Figure 3. After completion of the reaction, a strong and well-defined absorption peak at 540 nm suggested the formation of Py-AuNPs (Figure 3A). The kinetics of Py-AuNPs formation suggested that the formation of NPs began after 15 min of reaction with pyocyanin (Figure 3B) and was evident by observing the SPR signal characteristic of AuNPs at 540 nm (curve 1, Figure 3B). Initially, the SPR peak was broad, which gradually became sharper, suggesting NPs of uniform morphology were synthesized. After 60 min of reaction, no increase in SPR intensity was observed (curve 4, Figure 3B), indicating the completion of the reaction.

Representative TEM images of Py-AuNPs, obtained from reacting pyocyanin with the aqueous gold salt for 60 min, are shown in Figures 2C,D. A dense and uniform population of Py-AuNPs was present throughout the grid surface. At lower magnification large population of spherical PyAuNPs was observed without any aggregation (Figure 3C and Supplementary Figure 3A). The high magnification image (Figure 3D) of Py-AuNPs indicated that the average diameter of gold nanoparticles was around 12 nm with a standard deviation of ±0.92 nm and suggested that the particles were highly monodisperse (Supplementary Figure 3B). SAED analysis indicated the crystalline nature of Py-AuNPs and the diffraction pattern was indexed on the basis of the FCC structure of gold (Figure 3E). The corresponding *d* values for the respective crystal planes are as follows: 2.36 [111], 2.06 [200], 1.44 [220].

Figure 3F shows the FTIR spectra of Py-AuNPs before and after the NP formation reaction. The FTIR analysis before the reaction showed a number of bond vibrations corresponding to the various functional groups of pyocyanin (curve 1, Figure 3F). A few prominent vibrations were absent after the completion of the reaction and the formation of PyAuNPs. Particularly, a substantial variation was observed in the 1,500–1,700 cm^–1^ region of the spectrum. A region of the spectrum between 1,450–1,650 cm^–1^ corresponded to the stretching vibration of C = C of aromatic rings. The disappearance of a sharp absorption band at 1,509 cm^–1^ after Py-AuNPs formation could be due to the loss of the aromatic character of the pyocyanin ring structure (curve 2, Figure 3F). Also, a prominent absorption band at 1,603 cm^–1^ was shifted at 1,657 cm^–1^ indicating the possibility of alteration in the aromatic character of pyocyanin after reacting with chloroaurate ions (curve 2, Figure 3F). Out of plane C–H bond vibration of the aromatic ring present in pyocyanin was not observed after reaction with chloroauric acid (curve 2, Figure 3F). This observation also indicated that the aromaticity from pyocyanin ring structure could be altered after reaction with chloroaurate ions. The ring nitrogen of pyocyanin can donate one electron and get oxidized. This feature was observed in the FTIR spectrum, where a 3,058 cm^–1^ absorption band disappeared from the spectrum corresponding to pyocyanin after the reaction with chloroauric acid. Thus, from the FTIR spectrum, it can be concluded that the pyocyanin can cap the NP surface and stabilize them.

#### Synthesis and Characterization of Py-AgNPs

**FIGURE 4:**
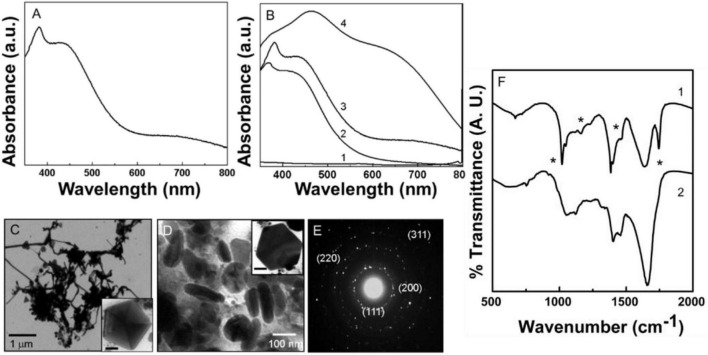
Uv-Vis spectroscopy, transmission electron microscopy (TEM), and Fourier transformed infrared spectroscopy (FTIR) analysis of biologically synthesized Py-AgNPs. **(A)** Uv-Vis spectra of Py-AgNPs showing the presence of the transverse and longitudinal SPR bands. **(B)** Py-AgNPs formation kinetics investigated by Uv-Vis absorption spectroscopy. Number 1-3 corresponds to 15, 30, and 60 min of reaction. **(C,D)** TEM images of Py-AgNPs were synthesized after 60 min of reaction. Inset in panels **(C,D)** show pentagonal and hexagonal population of nanoparticles. Scale bar in panels **(C,D)** is 50 nm. **(E)** SAED pattern obtained from Py-AgNPs showing crystalline nature of nanoparticles. **(F)** FTIR spectrum obtained from pyoverdine- silver ions reaction mixture before (1) and after (2) the formation of Py-AgNPs. Asterisks indicate the bond vibrations from the pyocyanin spectrum that are affected after NPs formation.

When silver ions were reacted with pyocyanin, the original colorless solution changed to yellow after 10 min of incubation, which eventually became yellowish brown after 30 min and finally dark brown after 60 min of the reaction. A Uv-Vis spectrum recorded from Py-AgNPs obtained after reduction of silver ions by pyocyanin is shown in Figure 4A. The reaction between pyocyanin and silver ions occurred at the comparable time scale of Py-AuNPs formation as discussed earlier. A characteristic SPR band at 420 nm was observed after 15 min of the reaction as indicated by curve 1 in Figure 4B. The SPR absorption around 420 nm was accompanied by a small sharp absorption peak at 390 nm, which slightly shifted to 405 nm after 30 min of reaction (curve 2, Figure 4B). However, this absorption band was not observed after 60 min of reaction (curve 3, Figure 4B). The initially observed SPR band at 420 nm showed a gradual increase in the intensity and a redshift with time. After 60 min of reaction, the initially positioned SPR at 420 nm showed an absorption maximum centered at 490 nm (curve 3, Figure 4B). The shift in the SPR absorption band from 420 nm indicated some degree of aggregation in newly synthesized silver nanoparticles. After 30 min of reaction, the SPR band at 440 nm was accompanied with a weak absorption at 680 nm (curve 2, Figure 4B). This absorption band showed an increase in intensity by 60 min of the reaction and can be observed either due to the presence of shape anisotropy or aggregation of nanoparticles, or both (29) (curve 3, Figure 4B). The band at 420 nm can be assigned to the transverse component and occurs due to the out-of-plane electronic oscillations, while the SPR band at 680 nm corresponds to the longitudinal component and originated due to the in-plane electronic oscillations in anisotropic AgNPs (29). No further increase was observed in the intensity of the SPR absorption band after 60 min of the reaction indicating that all silver ions were converted into AgNPs.

Figures 4C,D shows representative TEM images of Py-AgNPs after 60 min of reaction between silver ions and pyocyanin. A large number of NPs consisting of different morphologies like wires, triangles, pentagons, hexagons, and spindles were observed with some degree of inter-particle aggregation. The wire-like structures with a high aspect ratio were predominantly present among other morphologies. The wire-like structures were continuous and had interconnecting junctions (Figure 4D). The thickness of silver nanowires was 30 nm, and they grew in length up to 2 μm. A small fraction of spherical NPs appeared to be aggregated at the junction from where the nanowires branched out. The triangular and pentagonal morphology comprised a small population of NPs and showed the edge length between 100–300 nm (Inset Figures 4D,E). The triangular and pentagonal structures showed multiple twinning. A high magnification image of one of such pentagons clearly showed multiple twin boundaries in a five-fold symmetry (Inset in Figure 4E). The reduction of silver ions by pyocyanin also resulted in the formation of spindle-shaped Py-AgNPs, which were 100–200 nm long (Figure 4E). The SAED pattern obtained from Py-AgNps indicated the crystalline nature of nanoparticles and the diffraction pattern showed an excellent match with the FCC structure of silver. Respective *d* values for the corresponding crystal planes were: 2.374 [111], 2.05 [200], 1.44 [220], 1.24 [311].

Figure 4F shows the FTIR-spectroscopy analysis of Py-AgNPs before and after the reaction with pyocyanin. The FTIR profile of Py-AgNPs was very similar to Py-AuNPs suggesting a comparable redox mechanism for NPs formation. Most of the features of the spectrum obtained from Py-AgNPs (curve 2, Figure 4F) were comparable to the FTIR spectrum of Py-AuNPs (curve 2, Figure 3F) except for the distinct features in the 1,500 to 1,700 cm^–1^ region of the spectrum. The loss of a sharp absorption band at 1,509 cm^–1^, that was initially present in pyocyanin (curve 1, Figure 4F) could be due to the loss of aromatic character of the pyocyanin ring structure. Further, the wide absorption band at 605 cm^–1^, which corresponds to C = C bond vibrations in the aromatic ring was reduced in intensity (curve 2, Figure 4F). A sharp absorption band at 1,032 cm^–1^ in pyocyanin molecule corresponding to the C–O stretching vibrations (curve 1, Figure 4A), was shifted to a higher wavenumber and diminished in intensity after Py-AgNPs formation (curve 2, Figure 4F). This observation indicated a strong possibility for pyocyanin capping the surface of AgNPs. A sharp absorption band in the region 2,850 to 2,970 cm^–1^ assigned to the asymmetric and symmetric stretching vibrations respectively, arising due to the C–H bond in the CH_3_ group and the aromatic ring of pyocyanin (Supplementary Figure 4). This signal was considerably reduced after the synthesis of Py-AgNPs and appeared as a weak shoulder in the FTIR spectrum (Supplementary Figure 4). Supplementary Table 2 summarizes the vibration frequencies assigned for FTIR spectra shown in Figure 4F.

### Anticancer Activities of Pv-PNPs and Py-PNPs

**FIGURE 5:**
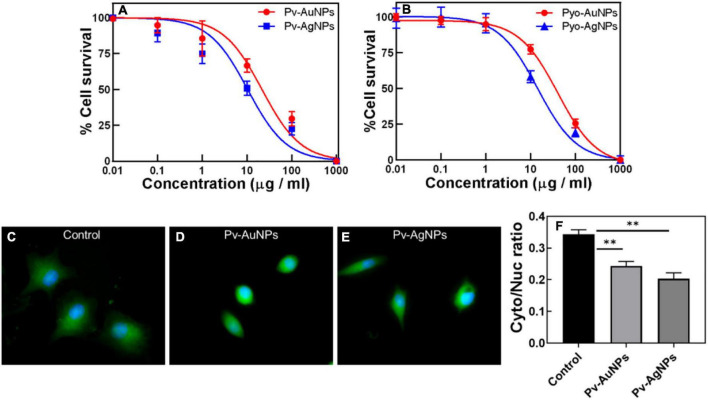
Anticancer activity of Pv-PNPs and Py-PNPs. **(A)** Cytotoxicity assay performed on A549 cells after treatment with Pv-AuNPs (red curve) and Pv-AgNPs (blue curve). **(B)** Cytotoxicity assay performed on A549 cells after treatment with Py-AuNPs (red curve) and Py-AgNPs (blue curve). **(C,D)** Effect of Pv-AuNps and Pv-AgNps on the cell and nuclear size in A549 cells. **(C)** Maximum intensity projection (MIP) of the composite image (overlay of polymerized actin and the nucleus) of untreated A549 cells showing well-defined cell body (green) and nucleus (blue) (Rai et al., 2021). MIP images of A549 cells treated with Pv-AuNPs **(D)** and Pv-AgNPs panel **(E)** showing reduced cell and nuclear size. **(F)** karyoplasmic ratio from control (untreated) and Pv-AuNPs and Pv-AgNPs treated cells showing reduction due to Pv-PNPs treatment (*P* = 0.0082 and 0.004). ^**^ indicates *p* value is statistically significant and is less than 0.01.

Considering cytotoxic and growth inhibitory properties of pyoverdine and pyocyanin, the effect of Pv-PNPs and PyPNPs was evaluated on metastatic lung cancer cells. The cell inhibitory potential against A549 cells was evaluated by performing MTT (3-(4,5-dimethylthiazole -2-yl)- 2,5- diphenyltetrazolium bromide) reduction cytotoxicity assay. Cytotoxicity measurement suggested that Pv-PNPs and PyPNPs were highly toxic to A549 cells. Figure 5 shows the effect of Pv-PNPs and PyPNPs on the survival of A549 cells. Pv-AuNPs and PvAgPs inhibited the growth of A549 cells by more than 75% at lower concentrations with respective IC_50_ values of 29.88 μg/ml and 10.33 μg/ml respectively (Figure 5A: blue curve- Pv-AuNPs; red curve-Pv-AgNPs). Though Pv-AuNPs and Pv-AgNPs showed similar cell inhibitory responses, Pv-AgNPs was more potent to inhibit the growth of A549 cells. Pv-PNPs were minimally toxic to cells at or below 10 μg/ml concentration, but a mere increase in concentration by two-fold showed an increase in the toxicity by many folds.

Py-PNPs also show a high cytotoxic response to A549 cells similar to Pv-PNPs. Figure 5B shows the inhibitory effect of Py-PNPs on the survival of A549 cells. Both Py-AuNPs and Py-AgNPs were highly toxic to cells at low concentrations with respective IC_50_ of 36.31 and 24.41 μg/ml. The relatively low IC_50_ of Pv-PNPs over Py-PNPs could be related to the metal ion chelating ability of Pv-PNPs due to the presence of pyoverdine on their surface. Also, it could be difficult for the cells to internalize long Py-Ag nanowires due to their morphology, and therefore, might show relatively reduced toxicity of Pv-AgNPs. The inherent cytotoxic ability of AgNPs increases manifold due to iron-chelating potential of pyoverdine and therefore Pv-PNPs were highly toxic to cancer cells.

**FIGURE 6:**
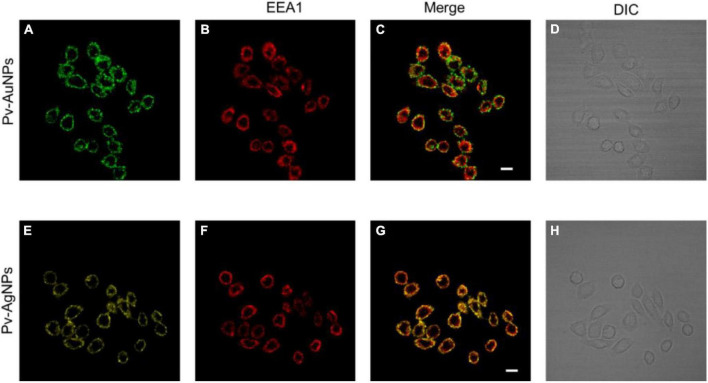
Internalization of Pv-PNPs in A549 cells. **(A–D)** Internalization of Pv-AuNPs **(A)** in A549 cells showing their presence in early endosomes **(B)**. Colocalization of Pv-AuNPs and EEA1 is shown by the overlay image **(C)**. **(D)** DIC image of A549 cells treated with Pv-AuPNPS. **(E–H)** Internalization of Pv-AgNPs **(A)** in A549 cells, and their localization in early endosomes **(F)**. Extensive colocalization of Pv-AgNPs in early endosomes is shown by the overlay image of Pv-AgNPs and EEA1 **(G)**. **(D)** DIC image of A549 cells treated with Pv-AgNPs. Scale bar indicates 20 μm.

Considering the high cytotoxicity of Pv-PNPs, we investigated the cellular uptake of these particles by fluorescence imaging. Pv-PNPs showed good fluorescence in the blue-green region that was detected with a confocal microscopy. A brief exposure (15 min) of Pv-AuNPs and Py-AgNPs to A549 cells resulted into their rapid uptake in endosomal vesicles (Figure 6). The fluorescence intensity associated with Pv-AuNPs and Py-AgNPs inside the cells did not differ significantly, suggesting equivalent uptake of Au and AgNPs by A549 cells (Figures 6A,E). Both Pv-AuNPs and Py-AgNPs were appeared to be preferably residing in early endosomes as indicated by merged fluorescence images (Figures 6C,G). This observation was further supported by the high colocalization of Pv-PNPs with EEA1 indicating their presence to be predominantly in the early endosomal compartment (Pierson’s correlation coefficient for Pv-AuNPs = 0.67; for Pv-AgNPs = 0.74) and possibly indicated for the same uptake mechanism of Pv-AuNPs and Pv-AgNPs in A549 cells. The elevated uptake of Pv-AuNPs, besides the shape anisotropy, could be the reason for their high toxicity to A549 cells.

Pv-PNPs treatment affected subcellular organization and altered the nuclear architecture of A549 cells. Figure 5B shows the effect of Pv-PNPs on cellular and nuclear organization before and after the treatment. A549 cells showed a well-defined morphology and the oval-shaped nuclei characteristic of the healthy state before the treatment of Py-PNPs (Figure 5C). After the treatment of Pv-AuNPs and Pv-AgNPs for 30 min, cells became more rounded and showed overall shrinkage in size. The nuclear architecture was altered and showed elongated and bulged morphology (Figure 5D), characteristic of a dying cell. The cells exposed to Pv-PNPs showed a reduced nucleocytoplasmic ratio compared to the untreated cells (Figure 5E). Pv-AgNPs treatment affected the nucleocytoplasmic ratio more drastically than Pv-AuNPs. Pv-PNPs treatment resulted in a 25 (for Pv-AuNPs) to 40% (for Pv-AgNPs) decrease in the nucleoplasmic ratio of A549 cells compared to untreated cells, suggesting for potent growth inhibitory effect of Pv-PNPs on cancer cells.

## Discussion

Green chemistry methods, involving the use of different living organisms and their products, have gained considerable attention as a viable alternative for the synthesis of nanoparticles. The biological synthesis of inorganic nanomaterials has been extensively investigated in the past couple of decades using microorganisms such as bacteria (Bansal et al., 2012), fungi (Hulkoti and Taranath, 2014), algae (Chaudhary et al., 2020), and various plant extracts (Mittal et al., 2013). Although aromatic plant extracts have remained a preferred choice for biological synthesis of anisotropic metal NPs (Shankar et al., 2004), bacteria and fungi are predominantly investigated for the production of complex inorganic nanoparticles such as metal oxides (Bharde et al., 2006), metal sulfides (Da Costa et al., 2016), and metal carbonates (Kim et al., 2015). While microorganisms have been extensively studied for nanoparticle biosynthesis, potential of their metabolites for the synthesis of the nanomaterial remains to be fully explored (Ben Tahar et al., 2019). Among various biosynthesis approaches, the synthesis of gold and silver NPs using a primary and a secondary metabolite obtained from Gram-negative bacteria *P. aeruginosa* is reported here. *P. aeruginosa* synthesizes many primary and secondary metabolites with excellent redox properties with the ability of single or multiple electron transfer reactions. Pyoverdine and pyocyanin are among such metabolites and show redox potential sufficient to reduce gold and silver ions subsequently resulting in NPs formation (Cezard et al., 2015). Additionally, the metal chelating ability of these metabolites makes them excellent capping and surface stabilizing agents as they can stably coordinate with the dangling metal ions present on the NP surface. Considering the cytotoxic activity of pyoverdine and pyocyanin, we further explored the potential of biologically synthesized PvPNPs and PyPNPs for anticancer activity. Biological methods though extensively researched for synthesis PNPs, are yet to be fully studied for anticancer activities.

*Pseudomonas aeruginosa* strain used as a pyoverdine and pyocyanin source in this study was obtained from a marine environment on the west coast of India (Roy et al., 2004). The bacterium showed inherent resistance to heavy metal ions, good salt tolerance, and efficiently degraded organometallic compounds such as tributyltin chloride. The isolate was identified as a strain of *P. aeruginosa* based on biochemical characterization and the 16 S rDNA molecular taxonomy method (GenBank accession number: DQ014538.1). The bacterium showed a strong green pigmentation when cultured on the King’s B medium and cetrimide agar suggesting the ability to synthesize pyoverdine and pyocyanin. It is likely that due to the adaptation to the marine environment, the bacterium evolved to produce pyoverdine and pyocyanin in higher quantities.

Purified pyoverdine and pyocyanin, when reacted with the gold and silver salts, rapidly yielded respective nanoparticles. Pyoverdine consists of a dihydroxyquinoline core linked to a small linear peptide containing 6–8 amino acids, most of them in a D configuration, and a ketoacid side chain (Peek et al., 2012). While pyocyanin is a small molecule that contains phenazine ring, and shows high redox activity. Both pyoverdine and pyocyanin show strong redox properties and participate in redox reactions involving mono or multivalent redox states (Cezard et al., 2015; Goncalves and Vasconcelos, 2021). Pyoverdine chromophore- amino dihydroxyquinoline carboxylic acid can undergo a two-electron transfer reaction. At the acidic or neutral pH, the carboxylic and hydroxyl group from pyoverdine can act as a nucleophile. The electrons donated by pyoverdine chromophore can be accepted by ionic gold and silver resulting in a stable metallic state. In addition, a peptide chain of pyoverdine with multiple ionizable hydroxyl and amine groups can donate multiple electrons as well. At the acidic pH, the hydroxyl groups from the peptide side chain can donate an electron and further enhance the reducing activity of pyoverdine. The low redox potential of pyoverdine (-0.04 V) (Cezard et al., 2015) can readily reduce gold and silver ions with a positive redox potential (1.5 and 0.8 V respectively). Pyoverdine, being an inherent metal chelator can also form dangling bonds with the valent metal ions present on NP surface through catechol and carboxylic groups, and help in capping and stabilization of the NPs.

Pyoverdine shows a higher affinity toward Au^3+^ ions and rapidly reduces them to Au^0^, eventually resulting in the formation of the triangular gold particles, as indicated by the Uv-Vis spectroscopy kinetics (Figure 1B). Formation of Pv-AuNPs was evident due to the well-defined SPR band just after 15 min of the beginning of the reaction. The resulting Pv-AuNPs show a high degree of shape anisotropy due to the presence of triangular and hexagonal particles. Interestingly, the edge length of Pv-AuNPs could be controlled by using the concentration of pyoverdine reacted with gold ions. It should be noted that pyoverdine reduced gold ions though resulted in triangular particles, spherical NPs were present in a noticeable amount, suggesting the need for further refinement of synthesis parameters to obtain a high yield of anisotropic NP population. FTIR spectra recorded before and after the formation of Py-AuNPs strongly suggested the role of –CO and –NH groups of pyoverdine in binding to NP surface for capping and stabilization purposes. Pyoverdine was also capable to reduce Ag^+^ ions and subsequently converting them into silver nanoparticles. However, the rate of silver ion reduction was slower initially (Figure 2B, curve 1), perhaps due to the lower affinity of pyoverdine to monovalent silver ions compared to trivalent gold ions. Pv-AgNPs did not show any shape anisotropy but a strong interconnected network of the particles that resulted into the broad SPR spectrum associated with scattering. Similar to Py-AuNPs, FTIR spectra of Py-AgNps suggested the role of –CO and –NH groups of pyoverdine in the capping and stabilization of NPs.

**FIGURE 7:**
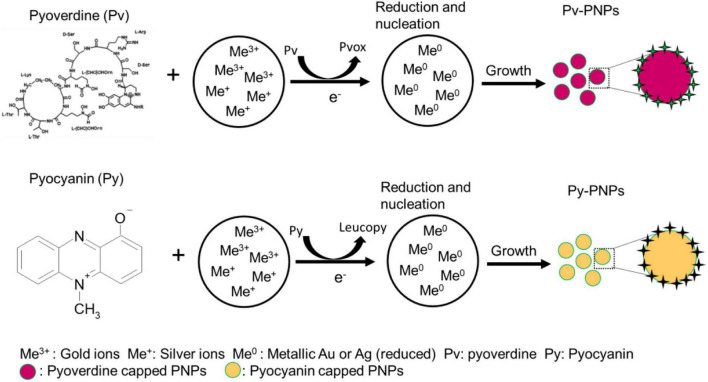
Schematics illustrating the pyoverdine and pyocyanin mediated reduction of gold and silver ions and subsequent formation of respective nanoparticles (Pv-PNPs and Py-PNPs).

Another metabolite explored in this study is a water diffusible pigment pyocyanin produced by *P. aeruginosa* during the stationary phase of growth in the bacterial life cycle. Pyocyanin consists of a phenazine ring and can exist in multiple redox states (Price-Whelan et al., 2007; DeBritto et al., 2020). It can act as a two-electron mediator redox switch and is capable of undergoing multiple redox cycles to form a colorless product leucopyocyanin, which in turn, is readily oxidized by the molecular oxygen. Additionally, it has a redox potential low enough (-0.034 V) (Price-Whelan et al., 2007) to reduce gold and silver ions. Due to its redox properties, pyocyanin can mediate numerous electron transfer cycles to gold and silver ions and convert them to metallic form. Like pyoverdine, pyocyanin shows a strong and moderate affinity for the multivalent and monovalent metal ions respectively. Therefore, it can bind to gold and silver ions present on the NPs surface and help in the stabilization of NPs in an aqueous environment. A schematic in Figure 7 illustrates the possible mechanism of gold and silver ion reduction and the formation of NPs.

Owing to its redox properties, pyocyanin enabled the reduction of gold and silver salts to form Py-Au and Py-AgNPs. Uv-Vis spectroscopic analysis of Py-AuNPs indicated that the formation of Py-AuNPs was rapid and resulted in a sharp SPR signal with a small integrated area under the curve, characteristic to the uniform-sized AuNPs (Basteus et al., 2014). Indeed, TEM analysis performed on Py-AuNPs showed the presence of monodispersed spherical nanoparticles with a very nano-size deviation (SD ± 0.9 nm, Supplementary Figure 3). FTIR spectroscopic analysis performed before and after the formation of PyAuNPs suggested the role of the ring nitrogen and methyl groups to participate in NP formation and stabilization respectively. Similar to the reduction of gold ions and Py-AuNPs formation, pyocyanin readily reduced silver ions to form Py-AgNps. Surprisingly, Uv-Vis spectroscopy suggested the presence of anisotropic nanostructures due to the presence of a two-component SPR signal. This was confirmed by TEM imaging, which showed numerous ribbon-like nanostructures with a few pentagonal and triangular nanoparticles. FTIR studies indicated a similar mechanism for the reduction of silver ions based on the absence of vibration patterns from Py-AgNPs. Although pyoverdine and pyocyanin show great promise for size and shape directed green synthesis of Au and AgNPs, the process was investigated in a small reaction volume (10 ml) and needs further investigations for scaling up operations Change in reaction volume from the test tube to a small reactor scale would require systematic investigations pertaining stoichiometric, kinetic and environmental parameters.

Considering the iron chelating ability of pyoverdine, we further explored Pv-PNPs for anticancer activity. Pv-PNPs were highly toxic to cancer cells and inhibit their survival at very low concentrations compared to other biologically synthesized Au and AgNPs (Zhang et al., 2020). The high toxicity of Pv-PNPs could be due to their rapid and greater uptake by cancer cells, which was evident by the presence of NPs inside the cells within a short time of incubation. Pv-AuNPs and Pv-AgNPs caused a 25 and 40% reduction in the karyoplasmic ratio of cancer cells compared with the non-treated control. Reduction in nuclear size suggested high genotoxic activity of Pv-PNPs, which could be due to the formation of reactive oxygen species due to the intracellular release of pyoverdine and metal ions from the NP surface. The anticancer activity of PNPs is strongly correlated with their physical traits such as shape (aspect ratio) and size (Chithrani et al., 2006). Uniform spherical NPs within the size range of 10–40 nm are generally rapidly internalized by tumor cells in high quantity compared to other sizes, and hence can be highly useful for anticancer drug delivery applications. Spherical PNPs also effectively sensitize tumor cells for radiation therapy and result in higher cellular destruction due to relatively large cellular uptake. While anisotropic NPs are significant for non-conventional treatment applications such as photodynamic therapy, owing to the longitudinal SPR component that strongly absorbs NIR radiation and effectively dissipates it as heat. Among various anisotropic NPs morphology, hexagonal, triangular, and star-shape NPs are highly effective due to the rapid heat dissipation from their pointed edges for the comparable aspect ratio (Barbillon, 2019).

Plasmonic nanoparticles, particularly AgNPs exhibit a strong redox activity and induce bursts of reactive oxygen species (ROS) production in a concentration-dependent manner. Both Au and AgNPs disrupt the redox homeostasis of cancer cells and elevate caspase activity that results in apoptotic death (Sukhanova et al., 2018). In our study, we did not observe a significant difference in cytotoxicity potential (IC_50_) between Pv-PNPs and Py-PNPs, the fact suggesting that all biogenic NPs perhaps act through a common growth inhibitory mechanism. The presence of redox-active metabolites such as pyoverdine and pyocyanin may further enhance intrinsic redox activity of AgNPs due to the leaching of surface ions, and augment ROS generating ability of the NPs. Metal ions released from the surface of AgNPs after cellular uptake can bind and denature many physiologically important proteins by oxidizing sulfhydryl groups. Moreover, AgNPs disrupt the electron transport process from the mitochondrial inner membrane and disturbs the mitochondrial redox potential. This results in rapid energy depletion and induction of apoptosis (Sukhanova et al., 2018). Since cancer cells have elevated energy demand for continuous growth and division, sudden disruption of energy supply due to mitochondrial dysfunction leads to rapid energy depletion and induction of apoptosis. Additionally, AgNPs interfere with the process of autophagy and promote the build-up of damaged mitochondria in the cell, further depriving the cells of energy production. AgNPs can also execute direct or indirect oxidative damage to genomic DNA and cause chromatin condensation and cell cycle arrest, which eventually lead to the rapid activation of apoptotic signaling and cell death. We believe, the presence of pyovedine on NPs surface significantly enhances their intrinsic antiproliferative potency to result in observed high cytotoxicity to cancer cells. Pyocyanin is a potent virulence factor and causes disruption in electron shuttling from the cytosol to mitochondria. It also interferes with normal mitochondrial activities vital for energy production and redox homeostasis, both vital for the cell survival. Pyocyanin presence at PNPs surface can rapidly disrupt cellular redox homeostasis leading to ROS generation that irreversibly damages cellular proteins and genomic integrity. These structural and functional damages subsequently lead to the activation of apoptosis. The acidic pH of the endosomal compartment may lead to the release of pyoverdine and pyocyanin from the PNPs surface and facilitate their transport to the cytosol. There, it can independently disrupt the redox homeostasis of the cell and help in enhancing apoptosis induction together, the ROS generation due to NPs. Besides chemical damage, Pv-AuNPs and Py-AgNPs can inflict structural damage to cellular components due to the anisotropic shape of these NPs. In the case of Py-PNPs, it is also possible for the scavenging of free iron ions by the pyoverdine molecules associated with the PNPs surface. Pyoverdine sequestration can limit the availability of iron, which is a vital micronutrient necessary for the growth and survival of cancer cells. However, the exact mechanism of Pv-PNPs’ enhanced toxicity to cancer cells requires further systematic investigations.

In summary, our results highlighted the potential of microbial metabolites for the shape and size-controlled synthesis of PNPs and the possibility of their use as a powerful anti-cancer agent. Due to the ability of pyoverdine and pyocyanin to rapidly form PNPs with appreciable shape and size control, it can be a preferred choice for PNPs synthesis. Although the biological synthesis of NPs is being explored with great interest due to its eco-friendly nature, it also suffers from significant drawbacks. Unlike wet-chemistry protocols, the biological synthesis of NPs using microorganisms is extremely slow. Additionally, it is complicated to control the experimental parameters, and hence predicting reaction output is difficult. Although phytoextract-based biological synthesis can kinetically compete with chemical synthesis methods, the inhomogeneous composition of the phytochemicals is a big challenge to minimize batch-to-batch variations and obtain uniform size and shape of NPs. Specialty applications for catalysis and optoelectronics require stringent control over the size and shape of the NPs, and biologically synthesized NPs may not be desirable for such applications in their current state. Though biological synthesis methods have yielded anisotropic NPs, control over the size and shape parameters such as aspect ratio, are far from realization compared to chemical or physical methods. Another drawback of biological NP synthesis is limited production capacity. It would be challenging to scale up the production of NPs using biological synthesis methods due to the complex nature of NPs synthesis (in case of microorganisms) and numerous unaccounted reaction parameters. The use of redox-active biomolecules can provide a better reaction control at par with chemical methods, compared to live microbial cells. However, this would require a systematic investigation to better understand the reaction stoichiometry, mechanisms of NPs synthesis, growth, and capping. Continuous advancement in biological synthesis methods would enable the tailored NPs synthesis and direct the possibility of large-scale production.

## Data Availability Statement

The raw data supporting the conclusions of this article will be made available by the authors, without undue reservation.

## Author Contributions

AB and MS conceptualized the work, designed the experiments, and interpreted the data. SP performed the experiments and helped in the data analysis. All the authors contributed in writing the manuscript.

## Conflict of Interest

The authors declare that the research was conducted in the absence of any commercial or financial relationships that could be construed as a potential conflict of interest.

## Publisher’s Note

All claims expressed in this article are solely those of the authors and do not necessarily represent those of their affiliated organizations, or those of the publisher, the editors and the reviewers. Any product that may be evaluated in this article, or claim that may be made by its manufacturer, is not guaranteed or endorsed by the publisher.
