# Supplementary material for: Size and Shape Directed Novel Green Synthesis of Plasmonic Nanoparticles Using Bacterial Metabolites and Their Anticancer Effects
Source: Front Microbiol. 2022 Apr 11;13:866849. doi: 10.3389/fmicb.2022.866849 (PMC9040069; doi:10.3389/fmicb.2022.866849)
Supplement: Supplementary file 1 [file Data_Sheet_1.PDF]

Size and shape directed, novel green synthesis of plasmonic nanoparticles using bacterial metabolites and their anticancer effects

Snehal Patil[1], Murali Sastry[2]*, Atul Bharde[1]*

[1]Department of Microbiology, savitribai Phule Pune university, Ganeshkhind, Pune 411007.

[2]Department of Materials Science and Engineering, Monash University, Clayton, Victoria 3800, Australia.

Supplementary information

Supplementary Figure S1: Charactrization of isolated pyoverdine (A) and pyocyanin (B) using thin layer chromatography. Both pyoverdine and pyocyanin show Rf values according to the published reports (see materials and methods for details). UV-Vis spectral characteristic of pyoverdine (C) and pyocyanin (D) showing absorption profiles characteristic to pyoverdine and pyocyanin respectively.

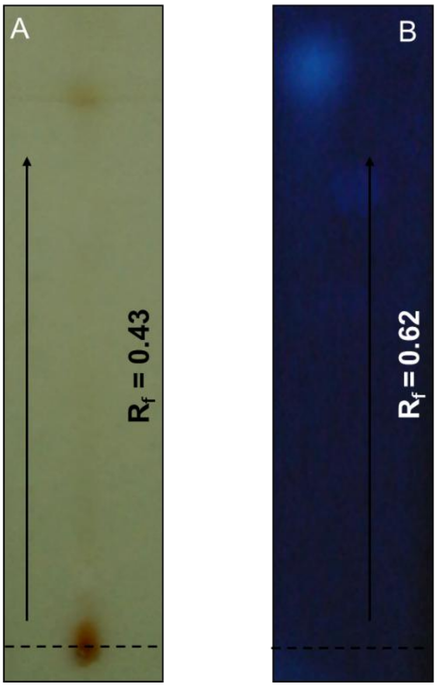

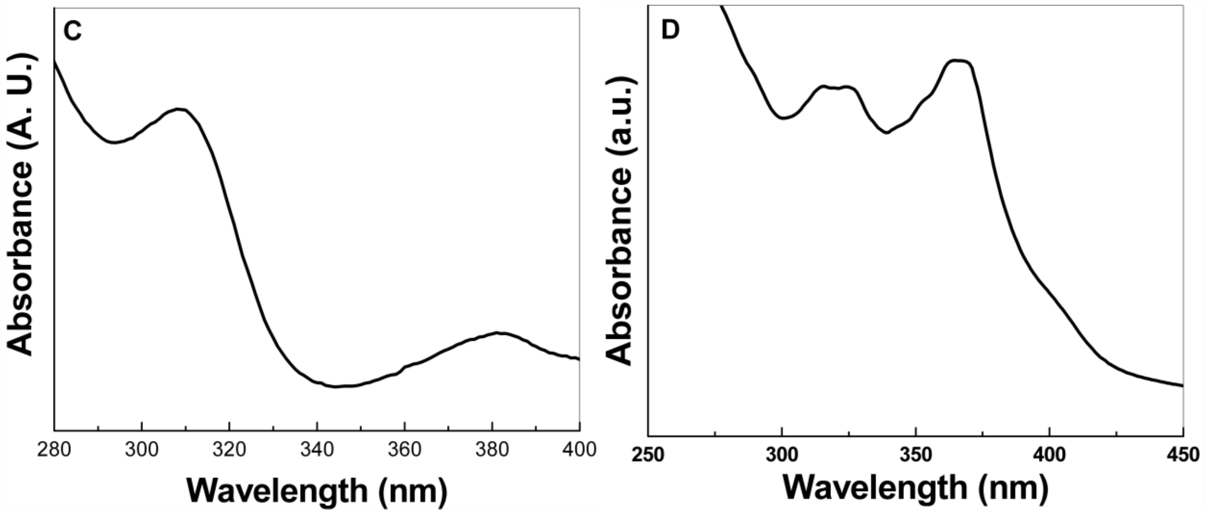
C
Absorbance (A. U.)
280
300
320
340
360
380
400
Wavelength (nm)
D
Absorbance (a.u.)
250
300
350
400
450
Wavelength (nm)

Supplementary Figure S2:

TEM images of Pv-AuNPs after reacting the low concentration (0.01 mg /ml) of pyoverdine with chloroauric acid for 3 h. Scale bar represents 500 nm.

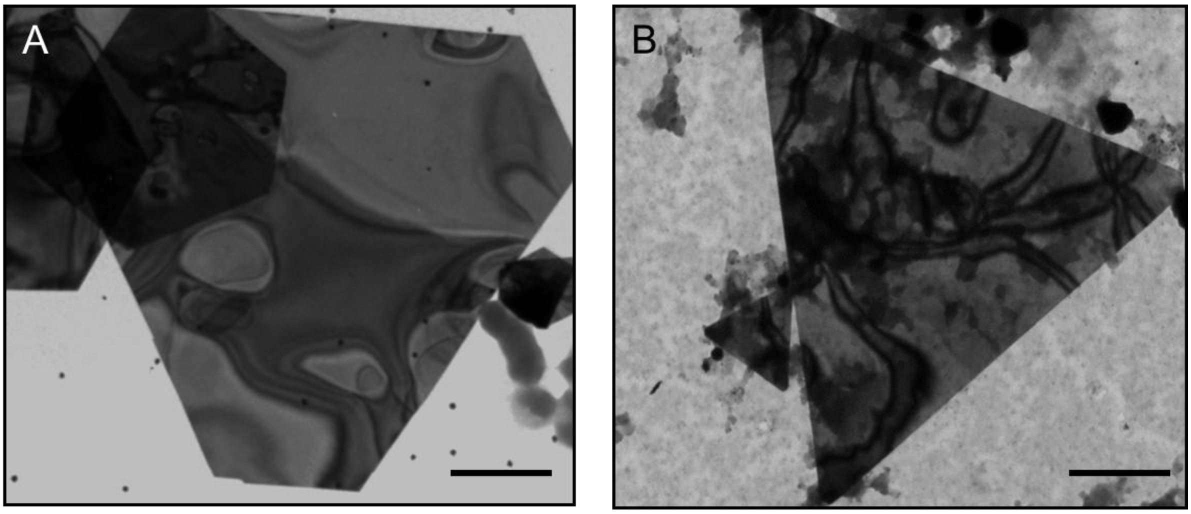

Supplementary Figure S3:

A low magnification TEM image of Py-AuNPs showing a large number of uniform sized NPs (A). The particle size distribution of Py-AuNPs shows a narrow size range. Most of the particles were between 12-14 nm in diameter as indicated by the frequency histogram (B) shown below.

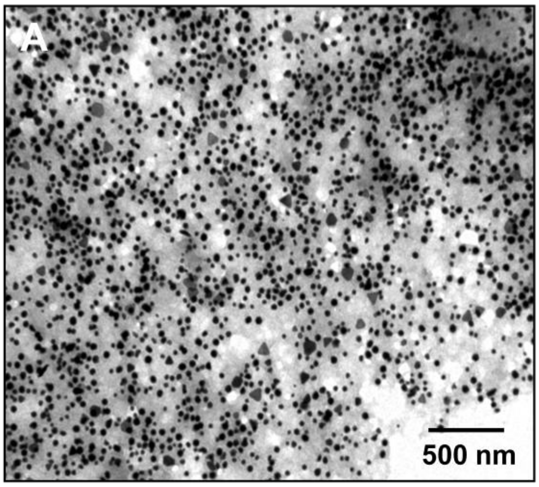

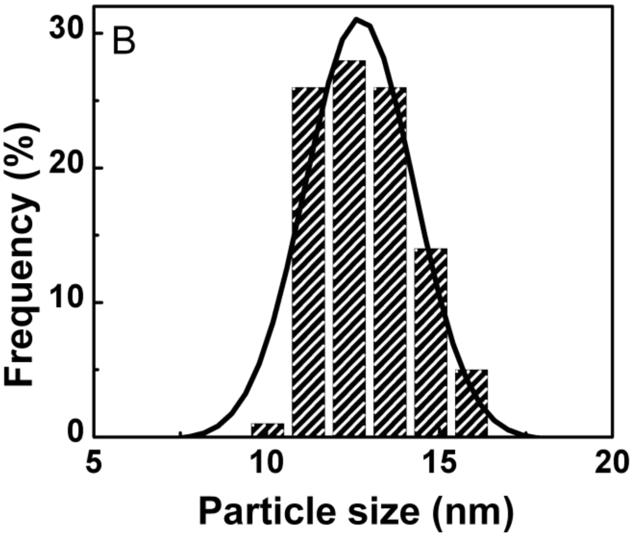

Supplementary Figure S4:

(A) The FTIR spectra (high wavenumber region) of pyoverdine before and after reaction with the aqueous chloroauric acid and the formation of Pv-AuNPs. (B) The FTIR spectra (high wavenumber region) of pyoverdine before and after reaction with aqueous silver ions and the formation of Pv-AgNPs. A vibration band characteristic to amine group of pyoverdine (curve 1) shows a large shift to low wavenumber after AuNPs formation (curve 2). While amine group associated with Pv-AgNPs show broad vibration spectra (curve 1) compared to pyoverdine (curve 1).

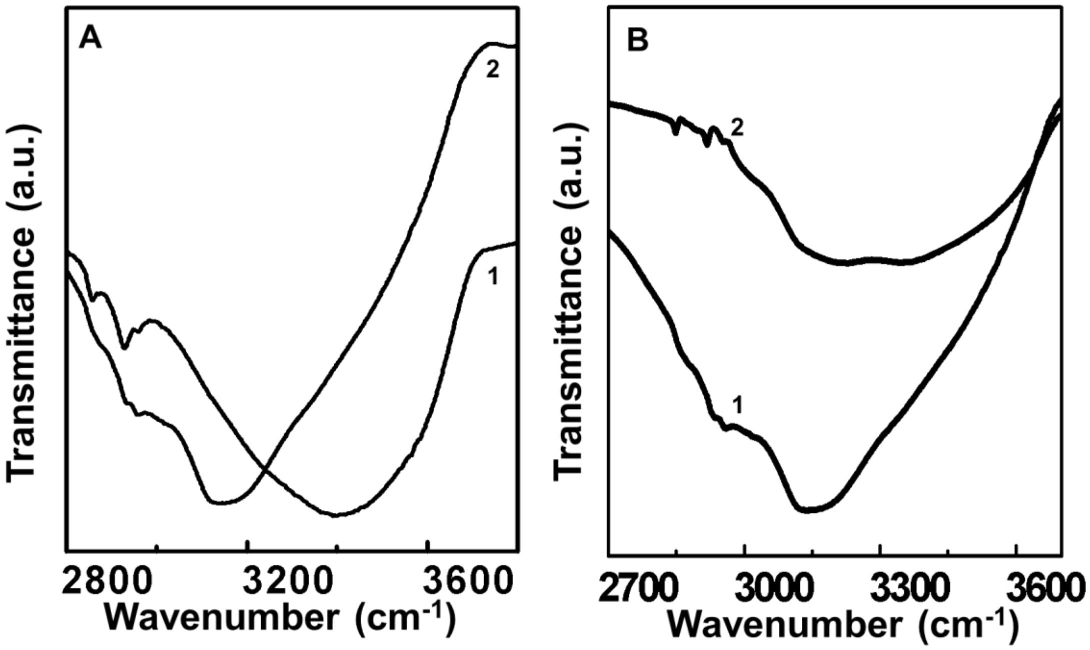

Supplementary Figure S5:

FTIR spectra (high wavenumber region) of pyocyanin befor and after reacting with gold and silver ions that resulted into the formation of Py-AuNPs (A) and Py-AgNPs (B). A prominent change in vibration pattern associated with the C-H stretching vibration is observed after formation of Py-AuNPs and Py-AgNPs.

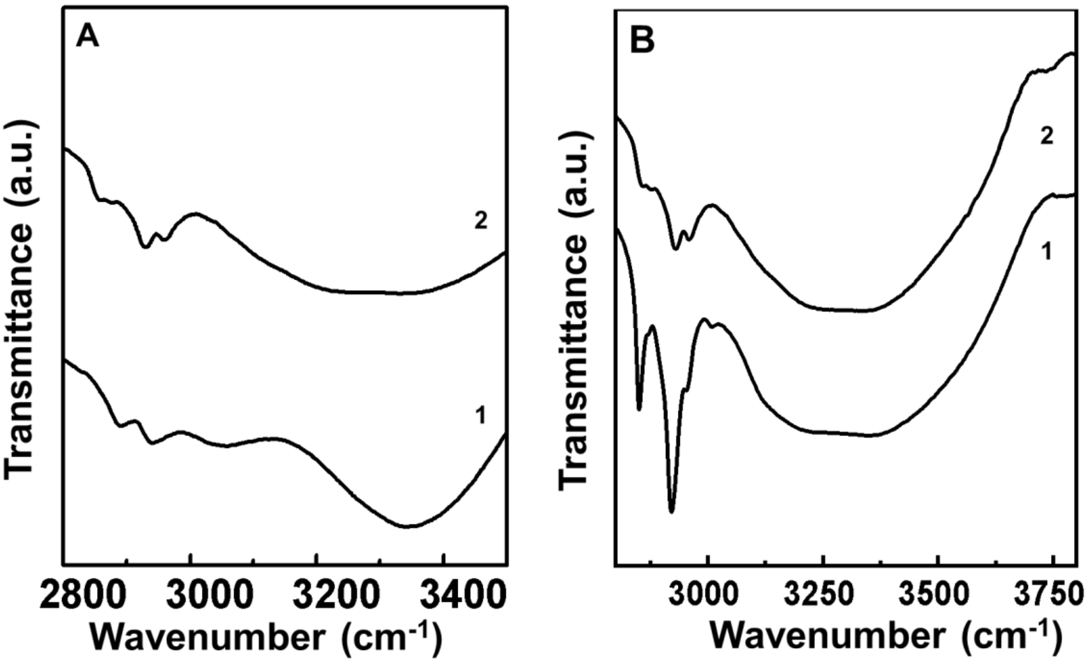

Supplementary table S1:

Vibration frequency assignment for the peaks observed in the FTIR spectra of pyoverdine before and after the reaction with gold and silver ions and formation of Pv-AuNPs and Pv-AgNps.

Vibration modes	Pyoverdin (cm^{-1})	Pv-AuNPs (cm^{-1})	Pv-AgNPs (cm^{-1})
C – O (ν)	1067	1048	--
C – N (ν)	1144	1142	1145
C – O (ν)	1221	– –	--
CH_3 (δ)	1402	1400	1405
O – C – H (δ), CH_2 (δ)	1438	– –	--
N – H (δ) of amide group	1545	– –	--
C = O (ν) of amide group	1663	1641	1645
C = O (ν) of aldehyde and ketone	1735	1730	1726
C – H	2929	2930	2925
N – H (ν) of amine group or O – H (ν)	3140	3400	3200
(δ) bending vibrations, (ν) stretching vibration			

Supplementary table S2:

Vibration frequency assignment for the peaks observed in the FTIR spectra of pyocyanin before and after the reaction with gold and silver ions and formation of Py-AuNPs and Py-AgNps.

Vibration modes	Pyocyanin (cm^{-1})	Py-AuNps (cm^{-1})	Py-AgNps (cm^{-1})
C – H of aromatic ring	896	– –	– –
C – O (ν)	1056	1060	1061
C – O (ν)	1120	1132	1130
CH_3 (δ)	1387	1400	1400
O – C – H (δ), CH_2 (δ)	1458	1470	1472
C = C of aromatic ring	1508	– –	– –
C = C of aromatic ring	1603	1657	1650
CH_3 (ν)	2895	2930	2900
C – H	3058	– –	– –
O – H	3344	3345	3350
(δ) bending vibrations, (ν) stretching vibration			
